# Laboratory diagnosis and treatment of *Mycoplasma pneumoniae* infection in children: a review

**DOI:** 10.1080/07853890.2024.2386636

**Published:** 2024-08-03

**Authors:** Li Gao, Yanhong Sun

**Affiliations:** Department of Clinical Laboratory, National Clinical Research Center for Child Health, National Children’s Regional Medical Center, Children’s Hospital, Zhejiang University School of Medicine, Hangzhou, China

**Keywords:** *Mycoplasma pneumoniae*, *Mycoplasma pneumoniae* pneumonia, laboratory diagnosis, treatment, children

## Abstract

*Mycoplasma pneumoniae* (MP) is the cause of *Mycoplasma pneumoniae* pneumonia (MPP) in children and adolescents, with the clinical manifestations highlighted by intermittent irritating cough, accompanied by headache, fever and muscle pain. This paper aimed to study the research status and focal points in MP infection, especially the common laboratory diagnostic methods and clinical treatment of *Mycoplasma pneumoniae*. Laboratory diagnostic methods include molecular assay, serological antibody detection, rapid antigen detection and isolation and culture. Polymerase chain reaction (PCR) is the gold standard with high sensitivity and specificity. The serological antibody can detect various immune antibodies qualitatively or quantitatively in serum. Rapid antigen can be detected faster, with no equipment environment requirements, which can be used for the early diagnosis of MP infection. While the culture growth cycle is long and insensitive, not recommended for routine diagnosis. Macrolides were the preferred drug for children with MPP, while the drug resistance rate was rising in China. Tetracycline can be substituted but was not recommended for children under 8 years of age, quinolone drugs are not necessary, severe MPP can be combined with glucocorticoids, involving the nervous or immune system can choose gamma globulin. Other treatments for MPP including symptomatic treatment which can alleviate symptoms, improve lung function and improve prognosis. A safe and effective vaccine needed to be developed which can provide protective immunity to children and will reduce the incidence of MPP.

## 1. Introduction

*Mycoplasma pneumoniae* (MP) is the smallest pathogenic microorganism, small prokaryotic cells without a rigid cell wall, which is between bacteria and viruses and can live independently, and the adhesion ability to host cells is positively correlated with virulence [1]. The genome size of MP is extremely small, about 816 kilo base-pairs [2]. 6 of the 16 species of human mycoplasma can cause diseases, and the most important and the most predominant pathogen is MP [3, 4]. The lack of a cell wall barrier in mycoplasma makes them insensitive to cell wall antimicrobials (such as beta-lactam), not stained by Gram staining, difficult to survive in dry environments, and also affects their appearance under the microscope [5]. MP attaches to ciliated cells within the respiratory epithelium *via* attachment organelles and produces an ADP-ribosyl transferase, also known as community-acquired respiratory distress syndrome toxin (CARDS toxin), which is responsible for entering host cells through clathrin-mediated endocytosis [6, 7].

MP can induce upper and lower respiratory tract infections, and cause *Mycoplasma pneumoniae* pneumonia (MPP), tracheobronchitis, etc., with headache, fever, muscle pain, sore throat, cough, dry cough or mucus-like sputum representing a predominant form of community-acquired pneumonia in pediatric populations, constituting a significant threat to children health [8, 9]. It can also cause various extrapulmonary manifestations, involving almost all organs, including skin and nerves, blood, cardiovascular, genitourinary system, musculoskeletal system, and can cause pseudomembranous necrotizing laryngotracheobronchitis, myelin oligodendrocyte glycoprotein antibody-associated meningoencephalitis [10–13]. Infections often occur in summer or early autumn, as well as at any time of the year. The main route of transmission is fulminant, the incubation period is 2–3 weeks, and the incidence rate is the highest among children and adolescents [14]. MP re-infects over some time probably because it can hide in host cells to protect it from antibodies and antibiotics; the second is the lack of protective immunity due to some important factors such as variation and rearrangement of surface antigens [15]. Studies have shown that clinical signs, symptoms and laboratory findings are not sufficient to distinguish pneumonia caused by MPP and other pathogens, and correct etiological diagnosis, as well as drug treatment largely depend on accurate and rapid laboratory diagnosis.

MP belongs to fastidious bacteria that makes culture difficult, and there is a considerable seropositivity rate in the population and the possibility of transient asymptomatic carriage, which makes serum antibody testing difficult to identify infected patients and normal people. The laboratory diagnosis of MP is challenging. Currently, the commonly used detection methods mainly include molecular assay (mainly polymerase chain Reaction (PCR)), serological antibody testing, rapid antigen testing and culture [16]. PCR includes real-time fluorescence quantitative PCR, nested PCR, reverse transcription PCR, and multiplex PCR. Serology is the basic strategy in routine diagnosis, and the results of different antibody classes are meaningful. Rapid antigen detection, especially the new colloidal gold immunochromatography, is a rapid, sensitive, and specific method for the detection of pulmonary branches. The culture of MP is too slow, and the sensitivity is low, so it is not recommended for routine detection. For the diagnosis of MPP, the pathogen and serological test results should be combined.

**Figure 1.**
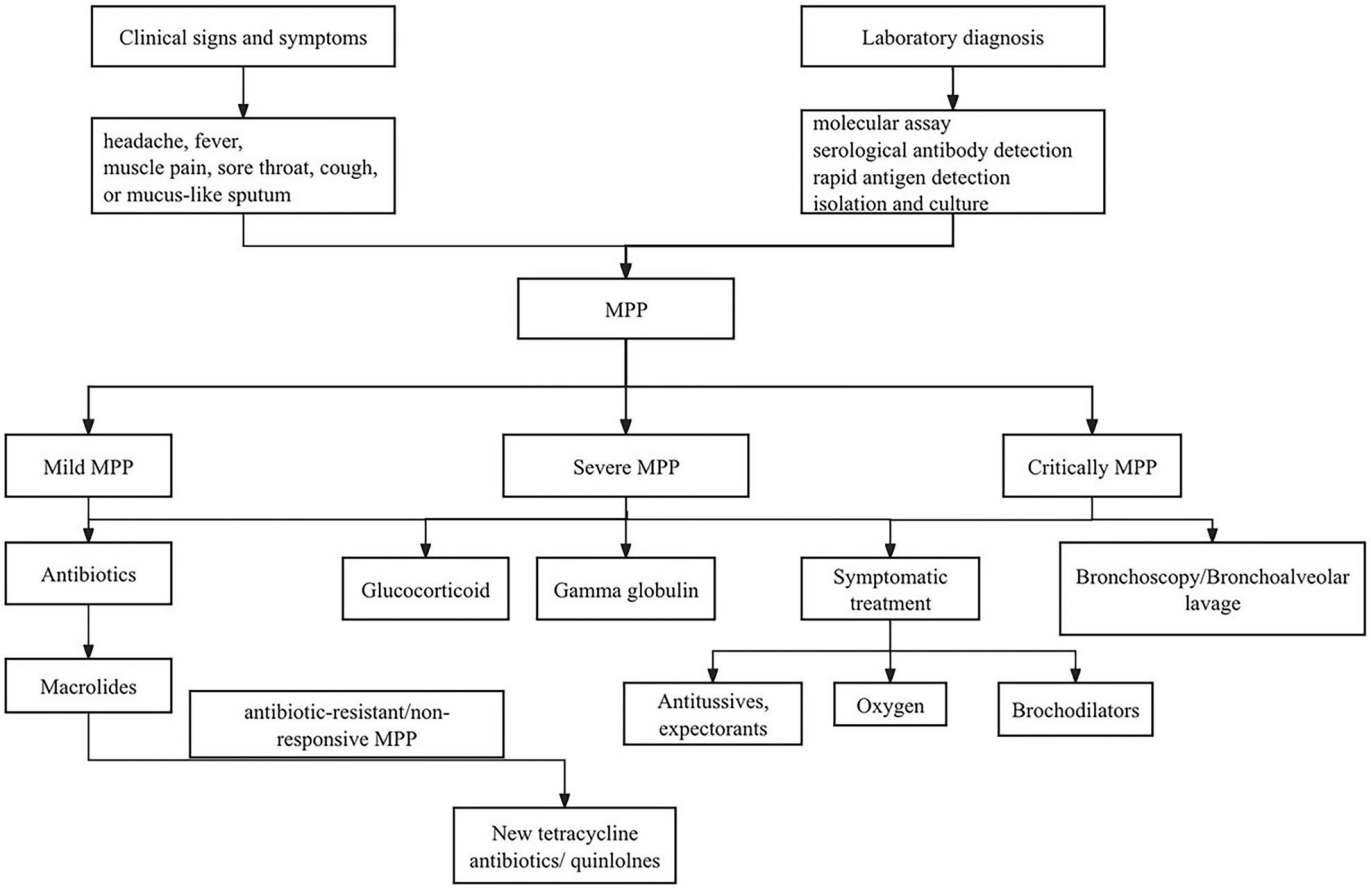
Flowchart of laboratory diagnosis and treatment of mycoplasma pneumonia in children.

MP infection is generally self-limited, and most mild patients can recover without treatment. The treatment of MPP mainly includes antibiotic therapy, glucocorticoids, immunoglobulin C and other combination therapy. Antibiotics mainly include macrolides, doxycyclines and new quinolones. The flowchart of laboratory diagnosis and treatment of mycoplasma pneumonia in children is in Figure 1.

## 2. Laboratory diagnostic methods

### 2.1. Molecular assay

PCR is considered the new "gold standard" with the higher sensitivity, most assays can detect <100 CFU/mL; The specificity is strong and there is no cross-reactivity when appropriate target selection and amplification conditions are validated. Nucleic acid amplification techniques used to detect MP DNA or RNA differ in the selection of target genes used (e.g. P1 gene, 16S rDNA, 16S rRNA, ATPase operon gene, etc.) (PCR versus isothermal amplification techniques) and the form of detection (conventional versus real-time, single versus multiple) [17]. The most problematic issue with PCR is colonization or asymptomatic carriage.

It is extremely rare to compare the performance of PCR methods with different *Mycoplasma pneumoniae* target areas and primers. P1 adhesin gene primers were found to be more sensitive than 16S rRNA primers, which may be due to the presence of multiple copies of the P1 cell adhesion gene. Studies have compared three different PCR detection methods: the detection method initially described by Bernet, with and without additional hybridization steps for amplicon detection, and the newly developed nested PCR [18]. All three PCR methods are reliable in detecting MP in respiratory specimens, but nested PCR is the most sensitive [19]. Due to the differences in sample collection, transportation and extraction procedures, input sample size, target genes, primers, cycle parameters, and detection systems, the comparison of sensitivity data for different PCRs becomes complicated.

A new detection platform MP-RPA-CRISPR for rapid, simple and accurate diagnosis of MP infection based on CRISPR-Cas12 b for recombinase polymerase amplification (RPA). The toxin gene of community-acquired respiratory distress syndrome (CARDS) was amplified by RPA, and the optimal reaction temperature was 37 °C. The amplified products were decoded by the CRISPR-Cas12b-based detection platform and interpreted by the real-time PCR system and the naked eye under blue light. MP-RPA-CRISPR can detect the genomic DNA template of MP strains, accurately distinguish MP strains from non-MP strains, and there is no cross-reaction [20].

DNA probes may be used for MP detection with 16S rRNA genes as the target, use a ^125^I-radioactive label to generate a detection signal, low sensitivity and specificity; rarely used at present [4].

Besides PCR, other alternative amplification techniques include nucleic acid sequence-based amplification (NASBA), Qβ replicase amplification, strand displacement amplification, transcription-mediated amplification and ligase chain reaction [21–24].

### 2.2. Serological antibody detection

After human infection with MP, MP-IgM, MP-IgA and MP-IgG antibodies can be produced. Specific serological detection is a common clinical diagnosis method at this stage and is mainly used to qualitatively detect various immune antibodies in serum or quantitatively detect antibody titers by immunization. Its sensitivity depends on the time point of collecting the first serum sample and the availability of paired serum collected at intervals of ≥ 2 weeks to evaluate serum conversion and/or antibody titer increase ≥ 4 times (“gold standard”) [25]. The interpretation of the qualitative results of the lung branch antibody is shown in Table 1. Specific serum immunoglobulin MP-IgM can usually be detected within about 1 week after clinical onset, and peak titer in the third week, which can be used as a diagnostic indicator of recent pulmonary infection. The increase, peak and decline time of specific serum MP-IgA were earlier than MP-IgM, which increased rapidly in the early stage of infection and had higher diagnostic accuracy [26]; MP-IgG antibody appeared late, which could be detected about 2 weeks after infection, peaked at 5 weeks and maintained for a long time. The low level but detectable MP-IgG antibody level may indicate the early stage of acute infection or previous infection, but it is not significant to detect MP-IgG alone [27]. In the case of low levels of specific MP-IgG, the second sample, as the recovery sample, must be collected after an interval of 2 weeks to prove that the antibody titer is significantly increased. Because the test is subjective, it is necessary to increase the titer of the double serum sample by at least 4 times to determine the diagnosis, which is the common standard for clinical diagnosis of pulmonary branch infection. The main disadvantage of MP-IgM positive alone in the diagnosis of pulmonary branch infection is that it cannot be produced continuously in adults, probably due to multiple previous infections, but MP-IgM positive is useful in the diagnosis of pulmonary branches in pediatric patients although IgM persists for a long time in children [28].

**Table 1.** Explanation of qualitative results of *Mycoplasma pneumoniae* antibody.

MP-IgM	MP-IgA	MP-IgG	Clinical significance
+	–	–	Recent infection
+	+	–	Present infection
+	–	+	Recent/Present infection
+	+	+	Recent/Present infection
–	+	+	Recent/Present infection
–	–	+	Past infection

Note: “+”: Positive result; “−” Negative result. Recent means within 1 or 2 years.

The quantitative detection results of pulmonary branch antibody titers are valuable for the diagnosis of the disease and the progression of the disease. The positive criteria are that the pulmonary branch specific antibody titer in the particle agglutination test is ≥ 1:160, or the pulmonary branch specific antibody titer in the complement binding test is ≥ 1:64, suggesting that the patient’s pulmonary branch is recent or current infection. In general, the serum antibody titer is positively correlated with the severity of the patient’s condition, which can provide a basis for clinical treatment and disease progression and recovery.

### 2.3. Rapid antigen detection

A new rapid antigen detection method was developed by using colloidal gold immunochromatography targeting the P1 gene region, with a detection limit of about 1 × 10^3^ colony-forming units (CFU)/mL. Compared with real-time fluorescence quantitative PCR, the specificity and sensitivity of the colloidal gold method were 100% and 97.4%, respectively [29]. Although the sensitivity is slightly lower than PCR, while the detection time is shorter, with no PCR equipment needed, only well-trained staff are needed, which has potential clinical application value in the early diagnosis of MP infection.

### 2.4. Isolation culture

The isolation and culture of the pulmonary branch are slow and insensitive and are not recommended for routine diagnosis, while a positive culture is 100% specific and irrefutable evidence of infection caused by MP. As the lung branch contains only a small group of enzymes, its pathogen growth requires higher nutrition, requires special enhanced broth or agar medium, and the growth cycle can be up to 3 weeks, with positive specimens sometimes being detected in just 5 days, but negative results need up to 6 weeks of incubation to confirm. The detection limit is 1 × 10^5^ colony-forming units (CFU)/mL, which is much lower than the sensitivity of PCR. Although the sensitivity of the lung branch culture is low, the isolation of pathogenic bacteria provides some understanding of the pathogenesis of the extrapulmonary system of lung branch infection and evidence of direct invasion of live mycoplasma. After identification and drug sensitivity test, it can provide a reliable basis for clinical diagnosis and drug sensitivity. It is worth noting that, like many other respiratory pathogens, MP can be detected in the upper respiratory tract of asymptomatic children. The detection rate of children without respiratory symptoms ranged from 3% to 56% [30–32].

### 2.5. Other new methods

Enzymatic amplification-free nucleic acid hybridization sensing on nanostructured thick-film electrodes by using covalently attached methylene blue allows the higher differentiation (with a 3.5 ratio) in the genosensing of M. pneumoniae [33]. A silver nanorod array-surface enhanced Raman Spectroscopy biosensing platform capable of detecting and distinguishing MP with statistically significant specificity and sensitivity in simulated and true clinical throat swab samples [34]. Recombinase-aided amplification (RAA) assay is a faster, sensitive and specific rapid detection method that has been used for the detection of MP, performed in a one-step single tube reaction at 39° Celsius within 15–30 min, with 100% sensitivity and 100% specificity [35]. A multiple cross displacement amplification (MCDA) coupled with a nanoparticle-based lateral flow biosensor (LFB) assay (MCDA-LFB) for rapid, simple and reliable detection of MP [36].

## 3. Clinical treatments

When MP infection is clearly defined, the main treatment method is drug treatment. Rational and standardized use of antibiotics can reduce symptoms and shorten the course of disease [37].

Mild MPP is more common in school-age children over 5 years old [38], with a course of 7–10 days, most patients have a good prognosis. The main clinical manifestations are fever and cough, wheezing and dyspnea can be detected in a small number of infants and young children. Imaging findings are bronchitis and bronchopneumonia; only a few patients can develop into severe [39]. Severe MPP refers to the severe condition of MPP, which conforms to any of the following manifestations: high fever ≥ 5 days or fever ≥ 7 days, or wheezing, shortness of breath, dyspnea, chest pain, hemoptysis and other symptoms. These manifestations are related to severe lesions, combined with plastic bronchitis, asthma attacks, pleural effusion and pulmonary embolism; extrapulmonary complications occurred, but did not meet the criteria for critical illness; finger pulse oxygen saturation ≤ 93% when breathing air at rest. The imaging findings were one of the following: large area of pulmonary consolidation; single lung diffuse or double lung multi-leaf segmental bronchiolitis showed [40, 41]. Critically MPP refers to severe MPP with rapid progression, respiratory failure or life-threatening extrapulmonary complications that require life-support treatment [42].

### 3.1. Antibiotics

MP lacks a cell wall and is resistant to all antimicrobials targeting the cell wall, susceptible to antibiotics that act on the bacterial ribosome and inhibit protein synthesis [43]. Commonly used antibiotics include macrolides such as azithromycin, clarithromycin, roxithromycin, etc. (Table 2), new tetracycline antibiotics such as doxycycline, minocycline and omarcycline, quinolones such as levofloxacin, ciprofloxacin, moxifloxacin. Tetracyclines can inhibit peptide chain lengthening of protein synthesis by acting on the 30 S subunit of MP ribosomes. The treatment time is generally 10 ∼ 14 days, and some severe patients can be extended to about 3 weeks [44].

**Table 2.** Antibiotics commonly used in MPP treatment.

Antibiotics	Macrolides	New tetracyclic antibiotics	Quinolones
	Azithromycin (second generation), clarithromycin, roxithromycin	Doxycycline (high safety), minocycline (strong effect), omalcycline	Levofloxacin, Ciprofloxacin, Moxifloxacin
Mechanism	binds to ribosomes in microbial cells, resists bacterial transpeptidase, and resists RNA-dependent protein synthesis.	specifically binds to the 30S subunit of bacterial nucleoprotein, prevents peptide chain extension and protein synthesis, and causes changes in cell membrane permeability, resulting in nucleotide leakage, thereby inhibiting DNA replication	Inhibition of bacterial DNA gyrase, so that DNA cannot form a spiral, cannot exist in the bacteria, inhibits DNA replication
Adaptation indications	Preferred drug, mild MPP	Alternative drugs for drug-resistant / non-responsive MPP, refractory and severe pneumonia	The effect is very good, but not necessarily not for children
Advantages	Strong tissue penetration, less gastrointestinal adverse reactions.	Effective for drug-resistant MPP.	Good effect on drug-resistant MPP, almost no drug resistance.
Recommended dosage and course of treatment	10 mg/kg once daily by oral/intravenous drops for 3 days	The first dose of minocycline is 4 mg/kg, not more than 200 mg, and the maintenance dose is 2 mg/kg after 12 h, twice a day, orally, for 10 days; Doxycycline is recommended at 2 mg/kg twice daily by mouth/intravenous drip	Levofloxacin for children from 6 months to 5 years old: 8–10 mg/kg twice daily, 5–16 years old: 8–10 mg/kg once daily, oral/intravenous drops, adolescents: 500 mg/d, qd, maximum dose of 750 mg/d, course of 7–14 days; Moxifloxacin 10 mg/kg once a day, oral/intravenous drops, for 7–14 days.
Adverse reaction	Gastrointestinal symptoms such as abdominal pain, bloating, nausea, vomiting and diarrhea; Elevated ALT, AST, allergic reactions such as drug rash and drug fever, allergies and temporary deafness, dermatitis, perineal erosion, shock, etc. Erythromycin can induce cardiotoxicity	Adverse effects on the growth and development of teeth and bones, resulting in yellowing of teeth and poor enamel development	Risk of cartilage damage and tendon breakage
Shortcoming	Drug resistance rates are getting higher and higher	Not suitable for children under 8 years of age	Not recommended for children under the age of 18
Attention	Close observation and electrocardiogram monitoring were performed during medication.	The use of off-label drugs in children under 8 years of age should be fully evaluated and parents informed consent should be obtained.	The use of off-label drugs in children under 18 years of age should be fully evaluated and parents informed consent should be obtained.

Macrolide antibiotics, represented by azithromycin and erythromycin, are the preferred drugs and have been widely used in children with MPP in recent years, while the drug resistance rate is also increasing, marked by point mutations in the 23S rRNA gene [45, 46]. Given the increasing prevalence of macrolide resistance worldwide, especially in East Asian countries such as Japan, China and South Korea, the search for alternative antibiotics to treat macrolide-resistance MPP is accelerating [47–49].

New tetracycline antibiotics including doxycycline, minocycline and omalcycline, are generally used for macrolide antibiotic-resistant/non-responsive MPP, refractory and severe pneumonia [44], with the main adverse reactions being tooth yellowing and enamel dysplasia, which were not suitable for children under 8 years old. Doxycycline is relatively safe for now due to a low affinity for calcium, and there is no report of tooth yellowing up to now when the recommended dose is used and does not exceed the course of treatment, usually 7–10 days [50, 51]. Tetracycline drugs are generally well tolerated, and common adverse reactions have been observed in patients receiving these drugs, including anorexia, nausea, vomiting, diarrhea, rash, photosensitivity and tooth discoloration [52]. Doxycycline regimens were shown to be more effective than macrolide regimens in MPP patients. The duration of fever and hospitalization was significantly shorter in patients with doxycycline regimens, and oral doxycycline is more acceptable to children [53]. The most concerning side effect of tetracycline drugs is permanent tooth discoloration, adverse reactions should be monitored at any time during treatment, especially tooth discoloration factors including dosage, duration of treatment, stage of tooth mineralization, and activity of the mineralization process [54].

Quinolones, represented by levofloxacin, ciprofloxacin and moxifloxacin, are not recommended for use in children under 18 years of age in China due to the risk of cartilage injury in some juvenile animals and tendon rupture in humans. In the 2011 guidelines for the treatment of community-acquired pneumonia in infants and children over 3 months of age, it is believed that quinolones can also be used for adolescents with mature bones or children ≥ 6 months who cannot tolerate macrolides. Quinolones, DNA synthesis inhibitors, defervescence can be achieved within 48 h [55]. Quinolones are more effective than macrolides in the treatment of macrolide-resistant MPP, and clinical improvement rapidly after receiving a fluoroquinolone in two patients with similar infections [56]. No reports of naturally occurring resistance to fluoroquinolones in MP up to now. After treatment with levofloxacin, the clinical symptoms and imaging significantly improved and no drug-related adverse reactions were observed [57]. Moxifloxacin is safe in the treatment of severe refractory *Mycoplasma pneumoniae* pneumonia (RMPP) in children according to a 31-children retrospective study [58]. *In vitro* activity against macrolide-resistant MP isolates has been reported for some fluoroquinolones, with MIC_90s_ being lower for moxifloxacin (≤0.0008–0.125 µg/mL) lower than for levofloxacin (0.25–0.5 µg/mL), and ciprofloxacin (0.5–4.0 µg/mL) [59–62]. Quinolones are generally well tolerated, with common side effects including gastrointestinal effects, headache and insomnia [63]. The potential risks of quinolones in pediatric patients should be considered carefully, particularly in Asia where resistance levels are high, as limited therapeutic options are available [62].

The fever of macrolide-resistant *Mycoplasma pneumoniae* infection lasted longer and was more severe, the blood oxygen saturation decreased, and the alanine aminotransferase (ALT) and lactate dehydrogenase (LDH) increased. Azithromycin combined with glucocorticoid may be a good treatment for macrolide-resistant *Mycoplasma pneumoniae* in children [64].

### 3.2. Glucocorticoid

For patients with rapid and severe disease progression in the acute phase, or patients with atelectasis, pulmonary interstitial fibrosis and bronchiectasis caused by RMPP, the ideal starting time of glucocorticoid treatment might be 5–10 days, after disease onset. The initial dose of glucocorticoid therapy should be determined according to the severity of the disease. High-dose glucocorticoid therapy may be required when MPP patients show total lobe consolidation on imaging [65, 66]. Commonly used drugs include methylprednisolone. A small amount of use has few side effects, and long-term large-scale application can easily cause adverse reactions [67, 68]. A randomized controlled trial finding three days of 2 mg/(kg·d) methylprednisolone therapy had an antipyretic effect in children with RMPP and could shorten the length of cough [69]. Other studies have found treatment with 2 mg/(kg·d) methylprednisolone can improve clinical symptoms and radiological manifestations of most children with RMPP quickly, but it may be ineffective in some situations [65]. Children with severe MPP may require a larger dose, and the changes of the children should be evaluated daily during the use period. If the high temperature drops significantly or becomes normal after 24 h of use, it is effective. If fever drops less than expected, insufficient dose, mixed infection or complications should be considered. The total course of treatment is generally not more than 14 days, if fever is repeated during the reduction process, it may be too fast, complications or drug fever [66, 70].

### 3.3. Gamma globulin

It is not a conventional treatment, only for the merger of central nervous system damage, immune system diseases such as immune hemolytic anemia, and immune thrombocytopenic purpura, consider the use of gamma globulin for adjuvant therapy. When children with MPP develop severe neurological complications, immediate intravenous administration of gamma globulin can lead to rapid and significant improvement in the clinical status of MPP-induced encephalitis [71].

### 3.4. Other treatments

Symptomatic treatment, includes giving small doses of antitussives and expectorants to relieve cough symptoms, giving oxygen to relieve hypoxia, and giving bronchodilators to improve asthma symptoms.

For RMPP, early flexible bronchoscopy has a significant effect. It can be combined with foreign body forceps to perform local irritation of the respiratory tract, remove secretions as soon as possible, avoid mucus blockage in the respiratory tract, or cause irreversible bronchial occlusion due to fibrotic contraction of the wall [72].

Bronchoalveolar lavage helps remove a large number of purulent secretions and sputum plugs in the bronchus of children with severe MPP, shortening the course of disease, and detecting pathogens through alveolar lavage fluid, which has a great guiding role in clear diagnosis and precise treatment, to improve the short-term and long-term prognosis and reduce the occurrence of complications [73]. Use with caution in patients suspected of pulmonary embolism.

Studies have shown that bronchoscope alveolar lavage (BAL) combination with budesonide, ambroxol + budesonide or acetylcysteine + budesonide in the treatment of RMPP can enhance the effectiveness of RMPP in children, increasing lung opacity absorption and minimize lung inflammation [74].

MPP can cause asymptomatic transient presence of lupus anticoagulant with isolated prolonged activated partial thromboplastin time [75]. Clinical reported the first pediatric case of splenic infarction following acute MPP with induction of anti-prothrombin antibodies, and reported aortic thrombus and multi embolisms during an MP infection, also reported severe hemolytic anemia and pulmonary embolism secondary to MPP [76–78]. With the improvement of the understanding of MPP, thrombosis in patients with severe MPP has become more common. In the whole treatment process of children, anticoagulant therapy cannot be ignored. Elevated serum D-D levels, specifically > 11.1 mg/L (even > 5.0 mg/L), would assist in the early diagnosis of thrombosis [79]. Increased levels of serum D-D can be used as an early predictor of RMPP and the occurrence of complications, which indicate excessive inflammatory response and vascular endothelial injury with prolonged duration in the patient population [80].

Vaccines for MP can protect children from infection, while human vaccine has yet to develop a vaccine suitable for general use, specifically targeting the emergence of macrolide-resistant MP. According to the pathogenesis of MP, researchers have conducted many immunogenicity studies, focusing on the whole cell antigen, adhesion protein antigen, and CARDS TX of MP, which laid the foundation for the final development of MP vaccine for human use.

## 4. Summary

The laboratory examination of MP is very important for identifying the pathogens of MPP, and the rational and safe use of antibiotics is also crucial for the treatment of MPP in children. MP is easily under-reported due to the lack of clinical and chest X-ray features, the relative lack of rapid and specific laboratory diagnostic techniques, and the difficulty of isolation and culture of MP. In most cases where the specific pathogen cannot be identified, doctors will give empirical beta-lactam antibiotic treatment, which is ineffective for atypical pathogens, and correct and timely use of macrolide antibiotics can significantly shorten the course of the disease, so a rapid and accurate laboratory diagnosis of MP is very important. The common laboratory diagnostic methods of MP include PCR, serological antibody detection, rapid antigen detection and isolation culture. PCR is the gold standard with high sensitivity, specificity and no cross-reaction. Serological antibody detection can qualitatively detect various immune antibodies in serum or quantitatively detect antibody titers, which have a certain guiding role in the diagnosis of the disease and the progression of the disease. The rapid antigen detection time is the fastest, and there is no equipment and environmental requirements, which can be used for the early diagnosis of MP infection. The growth cycle of isolation culture is long and insensitive, and it is not recommended for routine diagnosis. For mild MPP, macrolides are the first choice. For drug-resistant MPP, new tetracyclines and symptomatic treatment can be used instead, which will generally improve. For severe MPP, on the basis of symptomatic treatment and corresponding antibiotics, glucocorticoid and gamma globulin can be added, which can have obvious curative effects. For critically ill MPP, bronchoalveolar lavage can be added.

The diagnosis and treatment of MP are crucial in the entire disease process, especially for children. As pediatricians, we are most concerned about the diagnosis and treatment of a disease. The laboratory diagnosis method of MP may not be so accurate, and the treatment method is slightly weak due to the increase in drug resistance rate. The side effects of drugs in children need to be carefully considered during treatment. Our article combines these two parts, which is more suitable for pediatricians to have an overall understanding of MP, and thus, to better engage in clinical work.

## Data Availability

The data in this review are from published public domains literature, so data sharing is not applicable here as no new data were created or analyzed in this study.
